# Identifying Brief Message Content for Interventions Delivered via Mobile Devices to Improve Medication Adherence in People With Type 2 Diabetes Mellitus: A Rapid Systematic Review

**DOI:** 10.2196/10421

**Published:** 2019-01-09

**Authors:** Hannah Long, Yvonne K Bartlett, Andrew J Farmer, David P French

**Affiliations:** 1 Manchester Centre for Health Psychology School of Health Sciences University of Manchester Manchester United Kingdom; 2 Nuffield Department of Primary Care Health Sciences University of Oxford Oxford United Kingdom

**Keywords:** medication adherence, diabetes mellitus, systematic review, text messaging, mHealth, self-management

## Abstract

**Background:**

Current interventions to support medication adherence in people with type 2 diabetes are generally resource-intensive and ineffective. Brief messages, such as those delivered via short message service (SMS) systems, are increasingly used in digital health interventions to support adherence because they can be delivered on a wide scale and at low cost. The content of SMS text messages is a crucial intervention feature for promoting behavior change, but it is often unclear what the rationale is for chosen wording or any underlying mechanisms targeted for behavioral change. There is little guidance for developing and optimizing brief message content for use in mobile device–delivered interventions.

**Objective:**

This review aimed to (1) identify theoretical constructs (ie, the targets that interventions aim to change) and behavioral strategies (ie, features of intervention content) found to be associated with medication adherence in patients with type 2 diabetes and (2) map these onto a standard taxonomy for behavior change techniques (BCTs, that is, *active ingredients* of interventions used to promote behavioral change, to produce an evidence-based set of approaches that have shown promise of improving adherence in previous studies and which could be further tested in digital health interventions.

**Methods:**

A rapid systematic review of existing relevant systematic reviews was conducted. MEDLINE and PsycINFO databases were searched from inception to April 10, 2017. Inclusion criteria were (1) systematic reviews of quantitative data if the studies reviewed identified predictors of or correlates with medication adherence or evaluated medication adherence–enhancing interventions and included adult participants taking medication to manage a chronic physical health condition, and (2) systematic reviews of qualitative studies of experiences of medication adherence for adult participants with type 2 diabetes. Data were extracted on review characteristics and BCTs, theoretical constructs, or behavioral strategies associated with improved adherence. Constructs and strategies were mapped onto the BCT version 1 taxonomy.

**Results:**

A total of 1701 references were identified; 25 systematic reviews (19 quantitative reviews, 3 qualitative reviews, and 3 mixed-method reviews) were included. Moreover, 20 theoretical constructs (eg, self-efficacy) and 19 behavioral strategies (eg, habit analysis) were identified in the included reviews. In total, 46 BCTs were identified as being related to medication adherence in type 2 diabetes (eg, habit formation, prompts or cues, and information about health consequences).

**Conclusions:**

We identified 46 promising BCTs related to medication adherence in type 2 diabetes on which the content of brief messages delivered through mobile devices to improve adherence could be based. By using explicit systematic review methods and linking our findings to a standardized taxonomy of BCTs, we have described a novel approach for the development of digital message content. Future brief message interventions that aim to support medication adherence could incorporate the identified BCTs.

## Introduction

### Background

Diabetes mellitus affects an estimated 422 million people globally [1]. Approximately 90% of these cases are of type 2 diabetes [2,3]. Type 2 diabetes is typically managed through diet, physical activity, and oral medication. However, poor adherence to oral glucose-lowering medications in this population is common. The incidence of nonadherence is estimated to be between 38% and 93% [4-6], depending on the method used to define and measure adherence, and up to 37% of patients stop using oral glucose-lowering medications within 1 year of starting treatment [7]. This is a problem because medication nonadherence is associated with poorer clinical and health outcomes in patients with diabetes [8,9] and results in substantial financial burdens on Western health care systems because of higher rates of morbidity and mortality associated with the condition [10,11].

Effective interventions to increase medication adherence are needed to optimize health outcomes, quality of life, and cost-effective health care [12]. Despite the need, there is a lack of convincing evidence for interventions to improve patient adherence to prescribed medications for common chronic health problems. A Cochrane systematic review of 182 adherence-enhancing trials reported inconsistent effects on adherence, from highly heterogeneous interventions, and the majority of the lowest risk of bias trials (12 of 17) reported no improvements in adherence and clinical outcomes [13]. The interventions were generally multicomponent and complex, delivered by health care professionals and involving one-on-one education and counseling. Such interventions are time- and resource-intensive and, therefore, difficult to deliver to a large number of people or implement in usual practice settings. Similarly, complex interventions aimed at supporting medication adherence in patients with diabetes also report few improvements in adherence or clinical outcomes [14-18].

As of May 2014, 7 billion mobile phone subscriptions were in use worldwide [19]. In the United Kingdom, 65% to 92% of retired households own a mobile phone [20], and in 2017, 73% of adults accessed the internet through a smartphone or mobile device, which is double the rate recorded in 2011 [21]. The United States displays similar trends in increased mobile phone ownership; 77% and 95% of adults own a smartphone or mobile phone, respectively [22]. The increased use of mobile phone and modern technologies has created a new medium for digital health interventions delivered by mobile devices to support self-management of long-term conditions and potential solutions to problems such as medication nonadherence. Currently, the short message service (SMS) text message function of mobile phones offers a possible low-cost and wide-reaching means to deliver behavior change interventions. Brief messages delivered by SMS text message can be used in several ways: to send motivational and social support messages [23], to challenge maladaptive beliefs [24], or provide a cue to action [25]. This is important, as the impact on behaviors, such as medication adherence, is likely to differ depending on the message content.

A recent systematic review of brief messaging interventions for patients with type 2 diabetes concluded that these interventions may have the potential for supporting medication adherence [26]. However, the authors noted a widespread lack of explicit theoretical frameworks underpinning the included trials and called for greater use of theory in designing interventions to address the behavioral mechanisms through which changes to adherence may occur [26].

Behavior change theory can inform interventions by providing insight into factors that influence behavior, which can be targeted for behavioral change. This includes *theoretical constructs* (ie, those variables or constructs from theories that are targeted by interventions, eg, self-efficacy) and mechanisms underlying specific *behavioral strategies* (ie, techniques not necessarily linked to a single theory but incorporated in interventions because they predict behavior, eg, modeling). To translate these strategies into a standardized language, a comprehensive taxonomy of 93 *behavior change techniques* (BCTs) has been developed for use in behavior change interventions [27]. BCTs are discrete intervention components used to facilitate behavior change, such as problem solving and goal setting, and can be thought of as the *active ingredients* in interventions [27]. With this BCT taxonomy (BCTT), researchers can identify and evaluate specific techniques linked to intervention success, which facilitates the replication and optimization of existing or new interventions [27]. There is evidence that interventions that incorporate a greater number of BCTs tend to have larger effects on behavior than those incorporating a smaller number [28].

Grounding research in theory is crucial to progress understanding of which constructs need to change to, in turn, effectively change behavior. However, it is often unclear how message content has been developed, including what, if any, theory the messages are rooted in or how different types of messages may affect behavior. Without this, it is difficult to establish whether messages targeting constructs are effective or how to replicate these interventions. Toolkits for designing SMS-based interventions exist, but guidelines for developing the core message content are lacking (eg, The Center for Research in Implementation Science and Prevention [29]). An established process for developing brief message content, such as those delivered via SMS text message, is needed to optimize intervention effectiveness and facilitate evaluation of the most successful message types for changing behavior.

This review represents the first stage of a larger program of research to identify effective brief message content for use in interventions to support medication adherence in people with type 2 diabetes. We propose a novel approach to develop theory-based brief message content for use in interventions supporting medication adherence for people with type 2 diabetes. Given the lack of effectiveness of existing interventions [13], there is a need to determine promising novel intervention content for the future. For this reason, this rapid systematic review considered the factors related to adherence in 2 stages. First, we considered the factors found to affect adherence across a broad range of physical health conditions in previous systematic reviews of quantitative research. Second, we considered systematic reviews of qualitative research of adherence, specifically in people with diabetes to better understand the psychosocial context of and influences on adherence in our target population.

### Aims

The aims of this rapid review were to (1) systematically search for systematic reviews of quantitative studies that focused on medication adherence in physical health conditions and systematic reviews of qualitative studies that focused on adherence in type 2 diabetes, (2) extract data on theoretical constructs and behavioral strategies associated with medication adherence, and (3) map these constructs and strategies to BCTs to generate a list of BCTs that may show promise in improving medication adherence. We propose using these BCTs, to inform intervention targets and content of brief messages in future digitally based adherence-enhancing interventions.

## Methods

### Design

We conducted a rapid systematic review of systematic reviews. Rapid systematic reviews use methods to accelerate and streamline traditional systematic review processes while preserving the quality and rigor of review methods [30]. A rapid review is an appropriate method, given our aim to identify new potential intervention targets, rather than to conduct a comprehensive appraisal of the evidence base.

### Search Strategy

A systematic search of 2 electronic databases (MEDLINE and PsycINFO) was conducted from database inception to April 10, 2017. Search terms for medication adherence were chosen by adapting Medical Subject Headings (MeSH) and keywords previously used [13]. Search strategies were reviewed by the team. The search terms used in MEDLINE were “medication adherence [MeSH]” OR “medication compliance” OR “patient compliance [MeSH]” AND “review literature as topic [MeSH]” OR “systematic review” OR “meta-analysis” OR “meta-synthesis.” The search terms used in PsycINFO were “treatment compliance [MeSH]” OR “compliance [MeSH]” AND “literature review [MeSH]” OR “meta-analysis [MeSH]” OR “systematic review” OR “meta-synthesis.” EndNote reference software (Clarivate Analytics) was used to organize references.

### Inclusion and Exclusion Criteria

#### Scope of Included Reviews

Systematic reviews of quantitative data, qualitative data, or mixed-method data (reporting quantitative and qualitative data) were included. Only papers written in English were included. Systematic reviews of adult (aged older than 18 years) samples taking medication to self-manage a diagnosed physical health condition were included. Review samples with children and adolescents (aged less than 18 years) were excluded, as were samples from exclusively nondeveloped countries.

Systematic reviews of quantitative studies had to report on (1) interventions to improve patients’ medication adherence or (2) predictors of or correlates with medication adherence in physical health conditions. Systematic reviews had to report subanalyses of medication adherence if adherence was not the primary outcome measure. Reviews had to include a behavioral measure of medication adherence or a combination of behavioral and clinical measures. Reviews that reported only clinical measures of adherence were excluded, as these measures may be affected by behaviors besides medication adherence. Reviews had to report data on patient adherence in chronic physical health conditions. We excluded reviews of patient samples with severe mental health or psychiatric conditions, substance abuse, acute-only conditions, contraceptive or sexual function medication, herbal remedies, vitamins, vaccinations, homeopathy, or conditions that could be chronic (eg, cancer) but for which the treatment medication is short-term (eg, oral chemotherapy). Reviews of acute physical health conditions and nonsevere mental health conditions (eg, diabetes with comorbid depression or depressive symptoms) were eligible if they also reviewed chronic conditions.

Systematic reviews of qualitative studies of adult samples in which diabetes was the main health condition were included. Participants with other physical health conditions were eligible if diabetes was the majority health condition in the sample. To be eligible, mixed-method systematic reviews had to report findings in line with the above criteria for either the quantitative reviews or the qualitative reviews.

#### Theoretical Constructs and Behavioral Strategies

To identify individual theoretical constructs or behavioral strategies that were associated with medication adherence, reviews of quantitative studies had to report distinct constructs or strategies and their relationship with medication adherence through pooling data across studies. Reviews of quantitative studies were excluded if they did not breakdown interventions and studies into the separate components and examine the effects. However, reviews of quantitative studies that analyzed similar behavioral strategies under an umbrella term were included and assessed as a single strategy (eg, *self-monitoring of medication adherence* using different methods of self-monitoring). Reviews of quantitative studies were excluded if they examined only the effects of (1) the form of the interventions or studies (eg, intervention duration and interventionist), (2) sociodemographic variables (eg, ethnicity and gender), or (3) different lifestyle behaviors (eg, dietary intake and alcohol consumption) on medication adherence.

Systematic reviews of qualitative studies had to report patients’ experiences of, or barriers and facilitators to, taking and adhering to medication for type 2 diabetes.

### Data Extraction

Separate data extraction sheets were developed, piloted, and refined for quantitative and qualitative data. First, we extracted data from the systematic reviews of quantitative studies on a number of indicators (Multimedia Appendix 1) and the relationship between constructs and strategies with medication adherence.

Second, we extracted qualitative data in the form of the authors’ narrative results sections. Results pertaining only to patients’ experiences of adherence were extracted. The mixed-method reviews, and the data reported in these, were combined with either the pool of quantitative or qualitative reviews depending on whether the findings were more quantitative or qualitative in nature.

### Identifying and Mapping Theoretical Constructs and Behavioral Strategies to Applicable Behavior Change Techniques

There were 2 stages involved in the process of identifying and mapping theoretical constructs and behavioral strategies to BCTs. First, we used the reviews of quantitative research to identify foundational constructs and strategies that were positively related to adherence in a range of physical health conditions. For each of the constructs and strategies identified in the reviews of quantitative studies, a judgment was made about which BCTs in the BCTT may be an applicable technique to change that particular construct or strategy. This process was completed iteratively, reviewing and refining judgments to ensure that all constructs and strategies had been paired with at least one BCT, and any relevant BCTs were captured. The process of mapping involved a degree of brainstorming [31] and idea generation, a recognized early step in intervention mapping, with *the aim of generating as many explanations as possible in response to a question* [32]. We have adapted this approach, generating *answers*, in the form of BCTs, to the constructs and strategies (the *questions*) extracted from the reviews.

Second, we took a diabetes-specific approach and used the reviews of qualitative research to consider the specific context of our target population and to ensure that we did not overlook constructs or strategies that were particularly relevant to adherence in patients with diabetes. In this stage, a different method was used to identify constructs and strategies in the qualitative data and link these to BCTs. The reviews of quantitative studies, by nature, were generally explicit in the reporting of constructs or strategies, but these were implicit in the qualitative review data. Given this, to identify which constructs or strategies may be related to patient adherence, a reviewer first familiarized herself by reading and rereading the extracted data. Inductive line-by-line coding was conducted, taking into consideration the meaning and context of different barriers and facilitators to adherence. The codes applied were the constructs and strategies identified from the quantitative findings or concepts from relevant psychological theory. The reviewer then followed the same process of mapping to BCTs as that used for the quantitative review data.

The initial findings and mapped BCTs from the quantitative and qualitative data were discussed within the research team to provide a sense check and to draw from additional expertise. In light of this, the first author further refined the associations between constructs and strategies and BCTs.

## Results

### Search Results

**Figure 1:**
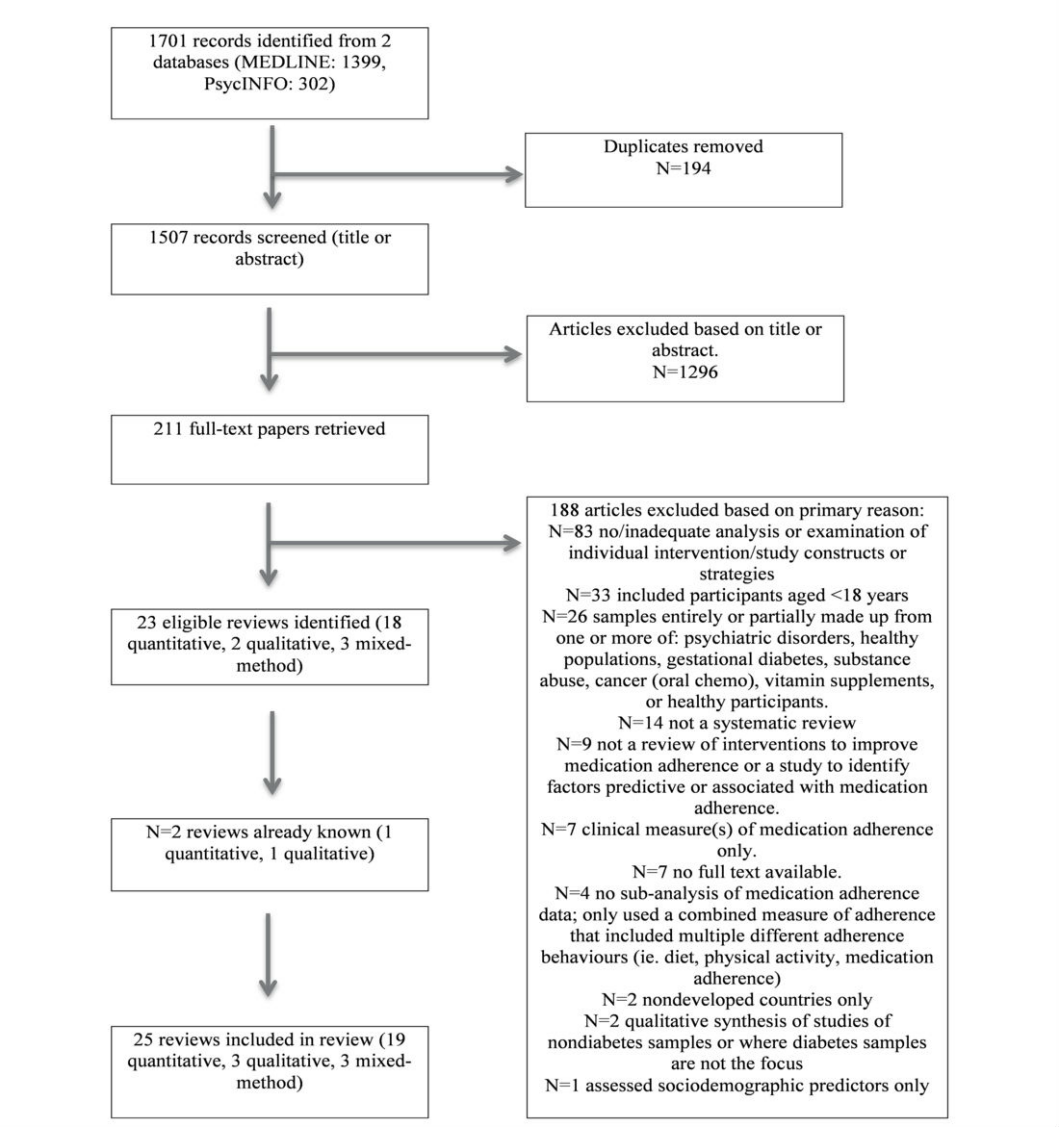
Flow diagram of study inclusion and exclusion process.

The electronic database searches identified 1701 references. After duplicates were removed, 1507 references remained. A reviewer screened all title or abstracts, and a second reviewer screened 29.86% (450/1507) of these (84% agreement). Moreover, 1296 references were excluded at the title-abstract stage. A reviewer read the full texts of the remaining 211 references, and the second reviewer read 29.9% (63/211) of these (71% agreement). Consensus on eligibility at both the title-abstract and full-text stage was reached through discussion. Any disagreements were resolved by discussing proposed reasons for inclusion and exclusion against the eligibility criteria. A total of 25 eligible systematic reviews (19 quantitative reviews, 3 qualitative reviews, and 3 mixed-methods reviews) published between 1995 and 2017 were included in this review—23 systematic reviews from database searches and 2 additional systematic reviews known to the researchers (Figure 1 illustrates the study inclusion process). From this point, 2 mixed-method reviews [33,34] have been integrated with the reviews of quantitative studies and the third mixed-method review [35] has been integrated with the reviews of qualitative studies.

### Systematic Review Characteristics

#### Systematic Review Aims

The reviews of quantitative studies examined interventions to promote medication adherence (k=16), predictive studies, or correlates of medication adherence (k=4) or both (k=1). The reviews of quantitative studies identified a total number of 1930 studies. The reviews of qualitative studies examined patients’ understanding of barriers and facilitators to adherence in type 2 diabetes (k=2), medication adherence–related beliefs and decision-making processes regarding taking medication in diabetes and cardiovascular disease patients (k=1), and patients’ perceptions and experiences of taking oral medication for type 2 diabetes (k=1). The reviews of qualitative studies identified a total number of 140 studies.

#### Patient Populations

Of the reviews of quantitative studies, 5 examined medication adherence in multiple chronic conditions and 4 reviews were of multiple chronic and acute conditions. The remaining reviews examined adherence in single conditions: hypertension (k=3), HIV and AIDS (k=2), type 2 diabetes (k=2), cardiovascular disease (k=1), chronic pain (k=1), heart failure (k=1), psoriasis (k=1), and organ transplantation (k=1). Overall, 2 reviews examined interventions targeted at health care providers to promote patient medication adherence. In addition, 3 reviews of qualitative studies included samples of diabetes patients, and the fourth review included a combination of samples of diabetes and cardiovascular disease patients.

#### Characteristics of Primary Studies

The primary studies in the reviews of quantitative studies were of various study designs; 14 reviews assessed multiple primary study designs. The study designs (when reported by review authors) included randomized controlled trials (RCTs; k=17), nonrandomized (k=8), pre- and posttest (k=4), prospective correlational (k=6), and cross-sectional correlational (k=5) designs (see Multimedia Appendix 1 for a summary table of review characteristics).

### Quantitative Systematic Review Findings

In total, 14 theoretical constructs and 13 behavioral strategies were extracted from 18 of 21 systematic reviews of quantitative studies because they were related to medication adherence (Table 1). There was variation in the methods used by review authors to analyze the relationship of a construct or strategy to medication adherence. The different approaches to analysis included moderation analysis, correlation analysis, calculating a tally of the number of primary studies that had found a statistically significant effect when a particular construct or strategy was present and, where possible, associated effect sizes, and qualitative comparative analysis to determine the necessity or sufficiency of an individual construct or strategy to influence medication adherence.

The 14 theoretical constructs and 13 behavioral strategies were mapped to 34 BCTs (Table 2). Each construct or strategy was linked with between 1 and 13 associated BCTs.

**Table 1:** Theoretical constructs and behavioral strategies associated with improved medication adherence extracted from the quantitative reviews.

Authors (year)	TC^a^ and BS^b^	Evidence summary
Broekmans et al (2009) [33]	Medication-related concerns (TC) Poor patient-physician communication and satisfaction (TC)	Concerns about side effects were a significant^c^ correlate of lower MA^d^ in k=1. Fewer concerns about withdrawal were a significant^c^ correlate of lower MA (k=1). Poor patient-physician communication and satisfaction were a significant^c^ correlate of lower MA (k=1).
Conn et al (2009) [36]	Coping with side effects (BS) Stimulus to take medication (BS) Self-monitoring of symptoms related to medications (BS) Providing succinct written instructions (BS)	Adding *side-effect management* as a moderator to a multiple-moderator model significantly^e^ improved the model (beta=.60). Interventions, including a stimulus to take medication were more effective at improving MA (ES^f^ 1.06) than interventions without these cues (ES 0.30). Interventions that directed participants to self-monitor symptoms related to medications (including symptom improvement from taking medications and medication side effects) were more effective (ES 1.18) at improving MA than interventions that lacked this component (ES 0.30). Interventions with succinct written instructions achieved better effects on MA (ES 0.61) than studies without succinct written instructions (ES 0.29).
Conn et al (2015a) [37]	No TC or BS associated with MA	N/A^g^
Conn et al (2015b) [38]	No TC or BS associated with MA	N/A
Conn et al (2016) [39]	Habit analysis (BS) Prompts or cues (BS)	Habit-focused interventions in which participants’ daily habits were linked to taking medications were more effective at increasing MA relative to interventions that lacked this component (0.57 vs 0.22^e^). Studies that used prompts or cues for taking medications had larger ES than studies that did not (0.50 vs 0.23^c^).
Conn et al (2017) [40]	Habit analysis (BS)	Moderation analysis showed that interventions that included habit analysis were more effective (*d*=0.37) at improving MA than interventions that did not (*d*=0.28).
Cutrona et al (2010) [41]	Reinforcement and reminding (BS)	The majority of k=16 showed small effects of reinforcement and reminding on MA, whereas k=3 yielded large effects.
Devine et al (1995) [42]	Self-monitoring of medications (BS) Self-monitoring of symptoms related to medications (BS) Increasing health-related knowledge through education (BS)	Effect size values on MA by type of treatment were monitoring medications (*d*=0.43), monitoring blood pressure (*d*=0.37), and education (*d*=0.81).
Dew et al (2007) [43]	Social support (BS)	Poorer social support was significantly associated with greater nonadherence (ES 0.10, CI 0.03-0.26^c^) from k=11.
Farmer et al (2015) [26]	Self-monitoring of medications (BS)	Overall, 3 of 6 self-monitoring trials observed significant^c^ improvements in MA.
Fogarty et al (2002) [34]	Social support (BS) Scheduling demands (TC) Regimen complexity (TC)	Social support was statistically significantly associated with MA in 1 of 4 papers and 1 of 8 abstracts. A total of 2 of 15 abstracts and 2 of 5 papers reported a significant^c^ association between scheduling demands and MA. Overall, 2 of 17 abstracts and 3 of 4 studies reported a significant association between regimen complexity and MA. In total, 2 of 4 of these found the direction of the association to be as expected; more complex regimens were associated with decreased MA.
Holmes et al (2014) [44]	Self-efficacy (TC) Perceived barriers (TC) Perceived adverse effects (TC) Perceived benefits (TC) Perceived severity (TC) Perceived susceptibility (TC) Attitude (TC) Intention (TC) Perceived behavioral control (TC) Necessity beliefs (TC) Medication-related concerns (TC)	Self-efficacy was a significant^c^ predictor of MA in 7 of 7 studies of sociocognitive theory, 6 of 6 studies of self-regulation theory, and 4 of 6 studies of social support theory. Perceived barriers were significantly^c^ associated with MA in 11 of 17 studies. Perceived adverse effects were significantly^c^ associated with MA in 4 of 5 studies. Perceived benefits were significantly^c^ associated with MA in 5 of 11 studies. Perceived severity was significantly^c^ associated with MA in 3 of 7 studies. Perceived susceptibility was significantly^c^ associated with MA in 3 of 6 studies. Attitude was significantly^c^ associated with MA in 2 of 5 studies. Intention was significantly^c^ associated with MA in 2 of 5 studies. Perceived behavioral control was significantly^c^ associated with MA in 2 of 4 studies. Necessity beliefs were significantly^c^ associated with MA in 7 of 8 studies. Medication-related concerns were significantly^c^ associated with MA in 7 of 8 studies.
Kahwati et al (2016) [45]	Self-efficacy (TC) Attitude (TC) Increasing health-related knowledge through education (BS) Motivational interviewing (BS)	Enhancing self-efficacy was identified as individually sufficient for improving MA (consistency 90%). Improving attitude was identified as individually sufficient for improving MA (consistency 90%). Increasing knowledge was a necessary individual BCT for improved MA; it was present in 31 of 34 studies (consistency 91%). Motivational interviewing was identified as close to the consistency threshold for an individually sufficient technique for improving MA (consistency 78%).
Ruppar et al (2015) [46]	No TC or BS associated with MA	N/A
Schedlbauer et al (2010) [47]	Reinforcement and reminding (BS)	In total, 4 of 6 studies reported statistically^c^ improved MA following reminders in the form of written postal material (k=1), regular telephone calls (k=2), and a simple calendar reminder of medication taking (k=1).
Simoni et al (2006) [48]	Interactive discussion of cognitions, motivations, and expectations about adherence (BS)	Interactive discussion of cognitions, motivations, and expectations about MA, ES 1.62 (CI 1.21-2.03; k=14) versus no discussion ES 0.99 (CI 0.55-1.79; k=4).
Takiya et al (2004) [49]	Prompts or cues (BS) Increasing health-related knowledge through education (BS)	Beeper: 1 of 1 study reported significant improvement in MA, ES 0.09 (CI −0.15 to 0.31^c^). Phone reminder: 1 of 1 study reported significant improvement in MA, ES 0.03 (CI −0.09 to 0.15^c^). Increasing health-related knowledge through education: 2 of 3 studies reported significant improvement in MA, ES 0.18 (CI −0.11 to 0.44^c^), 0.03 (CI −0.26 to 0.30^c^).
Teeter et al (2014) [50]	Motivational interviewing (BS)	Overall, 6 of 9 studies reported statistically significant^c^ differences between intervention and control groups for change in MA.
Thorneloe et al (2013) [51]	Patient satisfaction with their treatment (TC)	k=1 reported patients being too busy or fed up was associated with reduced MA.
Xu et al (2014) [52]	Tailoring care plan (BS)	Tailoring was the most common persuasive attribute; 76% of interventions that successfully improved MA included tailoring versus 33% of interventions in which MA did not improve^e^ (the number of included studies that incorporated tailoring was not reported).
Zomahoun et al (2015) [53]	Coping with side effects (BS)	Interventions in which *cope with side effects* was applied had a pooled SMD^h^ of 0.64 (95% CI 0.31-0.96) versus 0.02 (95% CI −0.25 to 0.28) for those who did not (the subgroup differences^e^).

^a^TC: theoretical construct.

^b^BS: behavioral strategy.

^c^*P*<.05.

^d^MA: medication adherence.

^e^*P*<.01.

^f^ES: effect size.

^g^N/A: not applicable.

^h^SMD: standard mean difference.

**Table 2:** Theoretical constructs and behavioral strategies identified by the reviews of quantitative studies and behavior change techniques mapped to these by the research team.

Theoretical constructs and behavioral strategies		Behavior change techniques
**Theoretical constructs**		
	Attitude	Framing or reframing, pros and cons, information about emotional consequences, information about health consequences, information about social and environmental consequences, and salience of consequences.
	Intention	Anticipated regret, comparative imagining of future outcomes, pros and cons, and verbal persuasion about capability.
	Medication-related concerns	Comparative imagining of future outcomes, framing or reframing, information about emotional consequences, information about health consequences, information about social and environmental consequences, problem solving, prompts or cues, pros and cons, reduce negative emotions, salience of consequences, social support - emotional, social support - practical, and social support - unspecified.
	Necessity beliefs	Anticipated regret, comparative imagining of future outcomes, framing or reframing, information about emotional consequences, information about health consequences, information about social and environmental consequences, pros and cons, and salience of consequences.
	Patient satisfaction with their treatment	Framing or reframing, pros and cons, and reduce negative emotions.
	Perceived adverse effects	Comparative imagining of future outcomes, framing or reframing, and incompatible beliefs.
	Perceived barriers	Framing or reframing, pros and cons, restructuring the physical environment, restructuring the social environment, social support - emotional, social support - practical, and social support - unspecified.
	Perceived behavioral control	Focus on past success, mental rehearsal of successful performance, self-talk, and verbal persuasion about capability.
	Perceived benefits	Anticipated regret, comparative imagining of future outcomes, information about emotional consequences, information about health consequences, information about social and environmental consequences, pros and cons, and salience of consequences.
	Perceived severity	Anticipated regret, comparative imagining of future outcomes, feedback on outcomes of behavior, framing or reframing, incompatible beliefs, information about emotional consequences, information about health consequences, and information about social and environmental consequences.
	Perceived susceptibility	Comparative imagining of future outcomes, framing or reframing, information about health consequences, and pros and cons.
	Regimen complexity	Habit formation, problem solving, and prompts or cues.
	Scheduling demands	Action planning and problem solving.
	Self-efficacy	Focus on past success, identification of self as role model, mental rehearsal of successful performance, self-talk, social reward, valued self-identity, and verbal persuasion about capability.
**Behavioral strategies**		
	Coping with side effects	Problem solving, social support - emotional, social support - practical, and social support - unspecified.
	Habit analysis	Behavioral practice or rehearsal, habit formation, habit reversal, and graded tasks.
	Increasing health-related knowledge through education	Information about antecedents, information about emotional consequences, information about health consequences, information about social and environmental consequences, and instruction on how to perform a behavior.
	Interactive discussion of cognitions and motivations and expectations about adherence	Anticipated regret, comparative imagining of future outcomes, framing or reframing, and pros or cons.
	Motivational interviewing	Comparative imagining of future outcomes, framing or reframing, pros or cons, and social support (emotional).
	Prompts and cues	Prompts or cues.
	Providing succinct written instructions	Instruction on how to perform a behavior.
	Reinforcement and reminding	Behavioral practice or rehearsal, habit formation, and prompts or cues.
	Self-monitoring of medications	Self-monitoring of behavior.
	Self-monitoring of symptoms related to medications	Self-monitoring of outcome(s) of behavior.
	Social support	Social support - emotional, social support - practical, and social support - unspecified.
	Stimulus to take medication	Prompts and cues.
	Tailoring care plan	Information about health consequences and social support (emotional).

### Qualitative Systematic Review Findings

In total, 17 theoretical constructs and 12 behavioral strategies were coded in the qualitative data [35,54-56] (Multimedia Appendix 2). Of these, 11 were not identified by the reviews of quantitative data, including 6 constructs: identity, motivation, negative emotions, response efficacy, social comparison, and social context and 6 strategies: credible source, demonstration of the behavior, problem solving, self-adjustment and experimentation with medication dose and frequency, self-management strategies, and self-monitoring of outcome(s) of behavior). The 17 constructs and 12 strategies were mapped to 46 BCTs; 12 BCTs were identified in addition to the 34 BCTs mapped from the quantitative review findings. Each construct or strategy had between 1 and 25 associated BCTs (Table 3).

### Selection of Candidate Behavior Change Techniques

In total, a pool of 46 BCTs were mapped from the theoretical constructs and behavioral strategies identified in the included reviews (Textbox 1, see Multimedia Appendix 3 for BCT definitions from the study by Michie et al [27]).

**Table 3:** Theoretical constructs and behavioral strategies identified in the reviews of qualitative studies and the mapped behavior change techniques from the behavior change technique taxonomy.

Theoretical constructs and behavioral strategies		Behavior change techniques
**Theoretical construct**		
	Attitude	Anticipated regret, framing or reframing, information about health consequences, and pros and cons.
	Identity	Identity associated with changed behavior, identification of self as a role model, incompatible beliefs, and valued self-identity.
	Medication-related concerns	Adding objects to the environment, anticipated regret, behavioral experiments, comparative imagining of future outcomes, conserving mental resources, credible source, demonstration of behavior, feedback on outcome(s) of behavior, framing or reframing, habit formation, information about emotional consequences, information about health consequences, information about social and environmental consequences, instruction on how to perform the behavior, monitoring of emotional consequences, problem solving, prompts or cues, pros and cons, reduce negative emotions, restructuring the physical environment, salience of consequences, self-monitoring of outcome(s) of behavior, social support - emotional, social support - practical, and social support - unspecified.
	Motivation	Anticipated regret, framing or reframing, salience of consequences, and self-talk.
	Necessity beliefs	Anticipated regret, behavioral practice or rehearsal, comparative imagining of future outcomes, feedback on outcome(s) of behavior, framing or reframing, information about emotional consequences, information about health consequences, information about social and environmental consequences, habit formation, pros and cons, salience of consequences, self-monitoring of behavior, and self-monitoring of outcome(s) of behavior.
	Negative emotions	Framing or reframing, information about emotional consequences, monitoring of emotional consequences, reattribution, reducing negative emotions, social support - emotional, and verbal persuasion about capability.
	Patient-physician relationship and communication	Credible source, framing or reframing, information about health consequences, social support - emotional, and social support - practical.
	Perceived barriers	Avoidance or reducing exposure to cues for the behavior, conserving mental resources, credible source, demonstration of the behavior, framing or reframing, habit formation, identification of self as a role model, information about antecedents, information about emotional consequences, information about health consequences, information about others’ approval, information about social and environmental consequences, instruction on how to perform the behavior, problem solving, prompts or cues, reducing negative emotions, restructuring the social environment, salience of consequences, social support - practical, social support - unspecified, and valued self-identity.
	Perceived behavioral control	Anticipated regret, behavioral practice or rehearsal, focus on past success, framing or reframing, information about antecedents, mental rehearsal of successful performance, reattribution, social support - unspecified, and verbal persuasion about capability.
	Perceived benefits	Anticipated regret, comparative imagining of future outcomes, feedback on outcomes of behavior, framing or reframing, incompatible beliefs, information about emotional consequences, information about health consequences, information about others’ approval, information about social and environmental consequences, and pros and cons.
	Perceived seriousness	Anticipated regret, comparative imagining of future outcomes, feedback on outcome(s) of behavior, framing or reframing, information about emotional consequences, information about health consequences, information about others’ approval, information about social and environmental consequences, and pros and cons.
	Perceived susceptibility	Anticipated regret, comparative imagining of future outcomes, information about health consequences, pros and cons, and reattribution.
	Regimen complexity	Conserving mental resources, habit formation, problem solving, and prompts or cues.
	Response efficacy	Anticipated regret, credible source, feedback on outcome(s) of behavior, information about health consequences, pros and cons, and self-monitoring of outcome(s) of behavior.
	Self-efficacy	Behavioral practice or rehearsal, feedback on outcome(s) of behavior, focus on past success, graded tasks, identification of self as a role model, information about others’ approval, mental rehearsal of successful performance, monitoring of emotional consequences, reduce negative emotions, self-talk, social reward, social support - emotional, and verbal persuasion about capability.
	Social comparison	Anticipated regret, comparative imagining of future outcomes, information about others’ approval, social comparison, social support - emotional, social support - practical, and social support -unspecified.
	Social context (support, influence, and stigma)	Avoidance or reducing exposure to cues for the behavior, credible source, demonstration of the behavior, generalization of a target behavior, identification of self as role model, incompatible beliefs, information about antecedents, information about health consequences, information about others’ approval, restructuring the social environment, social comparison, social support - emotional, social support - practical, social support - unspecified, and valued self-identity.
**Behavioral strategy**		
	Coping with side effects	Anticipated regret, information about health consequences, problem solving, social support - emotional, social support - practical, and reattribution.
	Credible source	Credible source.
	Demonstration of the behavior	Demonstration of the behavior.
	Habits	Action planning, behavioral practice or rehearsal, generalization of target behavior, graded tasks, habit formation, and habit reversal.
	Health-related information and knowledge	Action planning, credible source, information about emotional consequences, information about health consequences, information about social and environmental consequences, instruction on how to perform the behavior, reattribution, salience of consequences, and social support - practical.
	Problem solving	Action planning and problem solving.
	Prompts and reminders	Adding objects to the environment, prompts or cues, and restructuring the physical environment.
	Self-adjustment and experimentation with medication dose and frequency	Anticipated regret, behavioral experiments, comparative imagining of future outcomes, framing or reframing, generalization of a target behavior, information about health consequences, reattribution, problem solving, pros and cons, self-monitoring of behavior, and self-monitoring of outcome(s) of behavior.
	Self-management strategies	Behavioral practice or rehearsal, generalization of a target behavior, graded tasks, and habit formation.
	Self-monitoring of outcome(s) of behavior	Behavioral experiments, feedback on outcome(s) of behavior, self-monitoring of behavior, and self-monitoring of outcome(s) of behavior.
	Self-monitoring of symptoms	Information about health consequences, self-monitoring of behavior, and self-monitoring of outcome(s) of behavior.
	Tailoring care plan	Action planning, information about health consequences, graded tasks, and problem solving.

**The 46 behavior change techniques (grouped according to the behavior change technique taxonomy version 1) identified from systematic reviews of quantitative and qualitative studies as being promising candidates for future brief message interventions.**

1. Goals and planning

1.2. Problem solving

1.4. Action planning

2. Feedback and monitoring

2.3. Self-monitoring of behavior

2.4. Self-monitoring of outcome(s) of behavior

2.7. Feedback on outcome(s) of behavior

3. Social support

3.1. Social support (unspecified)

3.2. Social support (practical)

3.3. Social support (emotional)

4. Shaping knowledge

4.1. Instruction on ow to perform the behavior

4.2. Information about antecedents

4.3. Reattribution

4.4. Behavioral experiments

5. Natural consequences

5.1. Information about health consequences

5.3. Information about social and environmental consequences

5.2. Salience of consequences

5.4. Monitoring of emotional consequences

5.5. Anticipated regret

5.6. Information about emotional consequences

6. Comparison of behavior

6.1. Demonstration of behavior

6.2. Social comparison

6.3. Information about others’ approval

7. Associations

7.1. Prompts or cues

8. Repetition and substitution

8.1. Behavioral practice or rehearsal

8.3. Habit formation

8.6. Generalization of target behavior

8.4. Habit reversal

8.7. Graded tasks

9. Comparison of outcomes

9.1. Credible source

9.2. Pros and cons

9.3. Comparative imagining of future outcomes

10. Reward and threat

10.4. Social reward

11. Regulation

11.2. Reduce negative emotions

11.3. Conserving mental resources

12. Antecedents

12.1. Restructuring the physical environment

12.2. Restructuring the social environment

12.3. Avoidance or reducing exposure to cues for the behavior

12.5. Adding objects to the environment

13. Identity

13.1. Identification of self as role model

13.2. Framing or reframing

13.3. Incompatible beliefs

13.4. Valued self-identity

13.5. Identity associated with changed behavior

14. Self-belief

14.1. Verbal persuasion about capability

14.2. Mental rehearsal of successful performance

14.3. Focus on past success

14.4. Self-talk

## Discussion

### Principal Findings

This rapid review identified 25 published systematic reviews of medication adherence in patients with physical health conditions and extracted a total of 20 theoretical constructs and 19 behavioral strategies associated with medication adherence. These constructs and strategies were mapped to 46 applicable BCTs from the BCTT version 1 [27], which can be used in future medication adherence interventions. In the first stage, the reviews of quantitative research gave rise to 34 BCTs related to adherence in a broad range of chronic physical health conditions. To ensure the specific context of adherence in type 2 diabetes was accounted for, 12 additional BCTs were identified following review of the diabetes-specific qualitative data.

### Strengths

This review has several strengths. It is the first rapid review of systematic reviews that aimed to identify theoretical constructs and behavioral strategies related to medication adherence in patients with type 2 diabetes. We have followed established and explicit systematic review methodology for this rapid review; thus, minimizing bias in literature searching, retrieval, and appraisal [57]. Furthermore, taking a rapid systematic approach to synthesize an evidence base and using systematic review methods to consider existing systematic reviews are 2 relatively new approaches to evidence synthesis. In addition, this review proposes a novel approach to develop message content, which could be used to inform future brief message–based or other electronic health and mobile health (mHealth) interventions. Use of the methodology of this review may lead to the identification of promising BCTs for promoting alternative health behaviors, as it is likely that different BCTs will be relevant to different contexts. This has produced findings in line with the validated BCTT [27], a leading classification system by behavior change researchers. Finally, the use of theory is often missing or inadequately reported in interventions for enhancing adherence in patients with type 2 diabetes, which makes it difficult to interpret why, how, and where theory may have impacted intervention success [26]. This rapid review starts to address these issues, proposing why, how, and where to incorporate BCTs in brief message intervention design.

### Limitations

This review has several limitations. We used a simple search strategy to facilitate a rapid review. Although this was appropriate for our aims, forward and backward citation searching may have improved the comprehensiveness of the literature identified. A number of systematic reviews were excluded because the independent relationship between theoretical constructs and behavioral strategies and medication adherence were not reported, and instead, the focus was on establishing the overall effectiveness of generally complex interventions. The quality of the included reviews was not assessed and, therefore, the possible risk of bias in the included systemic reviews is unknown. However, eligible reviews had to have been conducted systematically to maintain a minimum requirement of methodological rigor. Furthermore, consensus on the definition, and essential methodological processes, of rapid reviews has not been reached [58-60] and the necessity of the quality assessment stage has been debated [30]. We adhered to a set of defining characteristics proposed for rapid reviews by recent research [30]. In a modified Delphi consensus study, and based on the opinion of 66 literature experts, Kelly and colleagues [30] concluded that rapid reviews (1) are conducted in a shorter time frame than systematic reviews (completed in up to 3 months, compared with an average 15 months for a full systematic review) [61-63], (2) use the most systematic and rigorous methods to synthesize evidence and answer the research question(s) as the time limit permits, (3) tailor methods typical of a systematic review to accelerate the review process, and (4) are transparent in reporting all methods and findings. By following these guidelines, we have produced a methodologically strong rapid review.

Our method of mapping was adapted from the intervention mapping approach and incorporated the subjective views of the authors. In future, primary research may benefit from using more rigorous and reproducible methods of mapping. This may be achieved through the use of established coding frames and mapping techniques derived more heavily from the intervention mapping literature. Valuable research in the area of mapping theoretical constructs to BCTs is underway and this should serve to progress and inform future endeavors of a similar nature [64]. However, there is currently no consensus on how to do this [64] and, as such, our approach was appropriate for our aim to identify a selection of BCTs that can be used as a basis for future research.

### Implications and Future Research

The primary practical contribution of this review is the identification of promising intervention content in the form of a set of 46 BCTs. These could be used by researchers to form the basis for developing brief messages in interventions to promote medication adherence in people with type 2 diabetes. Such interventions are a promising avenue for improving adherence [26]. Given the aims of our wider program of research, we advocate this particular application of our findings, but our findings are not limited to brief messages or populations with type 2 diabetes. The comprehensive list of BCTs could be incorporated into other modes of intervention delivery, with a variety of interactive capabilities, for example, mobile phone apps. In addition, the 34 BCTs mapped solely from the quantitative review findings may be applied more broadly to medication adherence in chronic physical health conditions, provided unique social and contextual influences on adherence were taken into account.

The primary research implication of this review is that it facilitates the development of a body of brief messages based on the identified BCTs. Each message could incorporate a BCT and use it to frame and support different aspects of adherence behavior in people with type 2 diabetes. It will be important to ensure that messages accurately reflect the BCTs with which they are associated. The process of content development may be improved by seeking expert input. This is the next planned step in our program of research; health care professionals and behavioral scientists will be asked to generate brief messages based on the target BCTs. These messages and associated BCTs will be further tested in people with type 2 diabetes to assess credibility and acceptability, and subsequently in behavioral scientists to assess fidelity. Following this, a feasibility trial and a full RCT will assess the effectiveness of the brief message intervention to support medication adherence in people with type 2 diabetes. The approach of this review may be particularly suited to the area of adherence, given that the need for new approaches has been identified by leading systematic reviews [13]. More broadly, other researchers may wish to adapt our approach to develop novel interventions for other behaviors or populations where there is a need.

This review is a starting point; we have cast a wide net to suggest new intervention content, given that existing interventions have been found to have little effect on medication adherence. Further research is needed to establish more precisely which BCTs might be most effective at supporting medication adherence in particular contexts as well as the factors that may influence effectiveness. In the context of diabetes, these factors could include health literacy, diabetes health competency, and numeracy. This is complex to establish, and such questions were beyond the scope of this review. When selecting BCTs, researchers should be guided by what they understand of the context and mode of intervention delivery and nominate BCTs that suit their particular setting, population, and research aims while considering any relevant disease-specific research. It will be important to achieve a balance between learning from previous evidence in the field and testing novel approaches to support adherence.

### Conclusions

This review has identified a range of theoretical constructs and behavioral strategies related to medication adherence and mapped these to 46 BCTs that may show promise in supporting adherence in people with type 2 diabetes. We propose developing and testing the effectiveness of brief message content in SMS text messages based on the 46 BCTs and have, thus, described a novel approach to designing the content of brief messages to optimize mHealth interventions.

## Appendix

Multimedia Appendix 1 Main characteristics of the included systematic reviews.

Multimedia Appendix 2 The coded qualitative data.

Multimedia Appendix 3 Behavior change technique definitions from Michie et al (2013) behavior change technique taxonomy version 1.

## Abbreviations

BCT: behavior change technique
BCTT: behavior change technique taxonomy
BS: behavioral strategy
ES: effect size
MA: medication adherence
MeSH: Medical Subject Heading
mHealth: mobile health
NIHR: National Institute for Health Research
RCT: randomized controlled trial
SMD: standard mean difference
SMS: short message service
TC: theoretical construct
