# Supplementary material for: Identifying Brief Message Content for Interventions Delivered via Mobile Devices to Improve Medication Adherence in People With Type 2 Diabetes Mellitus: A Rapid Systematic Review
Source: J Med Internet Res. 2019 Jan 9;21(1):e10421. doi: 10.2196/10421 (PMC6329430; doi:10.2196/10421)
Supplement: Multimedia Appendix 1 [file jmir_v21i1e10421_app1.pdf]

Multimedia Appendix 1

Table 1. Main characteristics of included systematic reviews.

	Authors (year)	K	Study design of primary studies	Patient populations	Medication adherence measures
**Quantitative reviews**					
	Conn et al., (2009) [36]	43	RCTs	Chronic and acute physical health conditions	Patient self-report, pill counts, electronic event monitoring system, pharmacy refill data
	Conn et al., (2015a) [37]	218	Healthcare provider targeted interventions including randomised and non-randomised treatment vs. control comparisons, treatment pre- vs. post-intervention comparisons, and control baseline vs. outcome comparisons.	Chronic and acute physical health conditions	Patient self-report, medication event monitoring systems, pharmacy refill data, healthcare provider-monitored adherence behaviour
	Conn et al., (2015b) [38]	101	Medication adherence interventions including randomised and non-randomised treatment vs. control comparisons, treatment pre- vs. post-intervention comparisons, and control baseline vs. outcome comparisons.	Hypertension	Patient self-report, electronic medication event monitoring systems, pharmacy refill data, pill counts
	Conn et al., (2016) [39]	53	Randomised and non-randomised medication adherence interventions	Chronic and acute physical health conditions	Patient self-report, electronic medication event monitoring systems, pharmacy refill data, pill counts
	Conn et al., (2017) [40]	771	Randomised and non-randomised treatment vs. control	Chronic and acute physical health	Patient self-report, electronic medication event monitoring systems, pharmacy refill data, pill

			comparisons in medication adherence interventions	conditions	counts
	Cutrona et al., (2010) [41]	82	RCTs	Cardiovascular disease (including hypertension, coronary artery disease, chronic heart failure, dyslipidaemia) and diabetes	Patient self-report, pill bottle caps
	Devine et al., (1995) [42]	102	Randomised, quasi-randomised, non-randomised, or pre-post single group	Hypertension	Patient self-report, prescription refill
	Dew et al., (2007) [43]	147	Cross-sectional and prospective correlational designs	Recipients of organ transplantation	Patient self-report including patient interview and survey, family and healthcare provider report collected via interview or survey, blood level measure, electronic medication monitoring, data from medical records
	Farmer et al., (2015) [26]	11	RCTs of interventions	Type 2 diabetes mellitus	Patient self-report including scales, questionnaires and dairies, prescription refill, redeemed prescriptions, returned medication blister packs, medication event monitoring system,
	Holmes et al., (2014) [44]	67	Prospective correlational and longitudinal designs	Chronic diseases including HIV, hypertension	Patient self-report, electronic monitoring systems
	Kahwati et al., (2016) [45]	60	RCTs	Chronic physical health conditions	Patient self-report, prescription fills and refills, medication event monitoring systems
	Ruppar et al., (2015) [46]	29	RCTs, quasi-experimental, non-randomised, controlled trial	Heart failure (comorbidities were common, such as hypertension, coronary	Patient self-report, pill count, electronic monitoring systems, pharmacy refill records

				artery disease, diabetes, and chronic respiratory disease)	
	Schedlbauer et al., (2010) [47]	11	RCTs	Cardiovascular disease	Pill count, prescription refill rate, electronic monitoring systems, patient self-report in diaries and interviews, physiological measures of tracer substances in blood or urine
	Simoni et al., (2006) [48]	19	RCTs	HIV and AIDS	Patient self-reported, electronic monitoring, physiological measure of viral load count
	Takiya et al., (2004) [49]	16	RCTs	Hypertension	Varied including self-report and pill counts
	Teeter &Kavookjian, (2014) [50]	9	RCTs, prospective correlational	Chronic physical illnesses	Patient self-report, health plan or pharmacy claims data
	Thorneloe et al., (2013) [51]	29	Cross-sectional correlational and longitudinal	Psoriasis	Patient self-report, pharmacy refill records, medication weights and counting, medication event monitoring system, physiological measures
	Xu et al., (2014) [52]	40	RCTs, non-randomised, pre- and post-test studies	Health conditions in older adults	Patient self-report, pill count, pharmacy refill records
	Zomahoun et al., (2015) [53]	14	RCTs, quasi-experimental, controlled pre- and post-test intervention studies	Type 2 diabetes mellitus	Patient self-report, medication event monitoring systems, prescription claims data
**Qualitative reviews**					
	Brundisini et al., (2015) [54]	86	Qualitative studies	Type 2 diabetes	n/a
	Kumar et al., (2016) [55]	21	Qualitative studies	Type 2 diabetes and cardiovascular disease	n/a
	McSharry et al., (2016) [56]	8	Qualitative studies	Type 2 diabetes	n/a
**Mixed-method reviews**					

	Broekmans et al., (2009) [33]	14 (13 quantitative, 1 qualitative)	RCTs, prospective correlational, cross-sectional correlational, qualitative studies	Chronic pain, chronic headache/migraine, osteoarthritis, rheumatic diseases, fibromyalgia	Patient self-report, electronic monitoring, pill count, pharmacy refill records, physiological measures
	Fogarty et al., (2002) [34]	94 (88 quantitative, 5 qualitative, 2 mixed-method)	Cross sectional correlational, prospective longitudinal correlational, studies with qualitative data	HIV and AIDS	Patient self- or other-report, pill count, pharmacy refill records, mechanical and electronic monitoring, physiological measures
	Ng et al., (2015) [35]	25 (15 qualitative, 10 quantitative)	Qualitative research studies, cross-sectional correlational, case-control	Type 2 diabetes	Self-designed questionnaires

AIDS acquired immunodeficiency syndrome

HIV human immunodeficiency virus

RCTs randomised controlled trials
