# Supplementary material for: Identifying Brief Message Content for Interventions Delivered via Mobile Devices to Improve Medication Adherence in People With Type 2 Diabetes Mellitus: A Rapid Systematic Review
Source: J Med Internet Res. 2019 Jan 9;21(1):e10421. doi: 10.2196/10421 (PMC6329430; doi:10.2196/10421)
Supplement: Multimedia Appendix 2 [file jmir_v21i1e10421_app2.pdf]

Multimedia Appendix 2

Qualitative data extracted from the three systematic reviews of qualitative studies and one mixed-method review, and the constructs and mapped BCTs.

Table 1. McSharry et al. (2016).

Quote	Construct	Possible BCTs
Theme: Medication for diabetes: a necessary evil		
There was a 'resounding view that drugs were beneficial'	Necessity beliefs (medication beneficial)<br><br>Perceived benefits	Salience of consequences<br><br>Anticipated regret
Patients appreciated the need for medication, felt lucky to have it and perceived short- and long-term benefits of medications. Specific benefits of oral medications included controlling blood glucose, preventing complications, and staying healthy and increasing physical well-being	Perceived benefits<br><br>Response efficacy	Information about health/social/emotional/ environmental consequences, salience of consequences.<br><br>Information about health consequences, feedback on outcomes of behaviour, credible source
Disadvantages and risks of medications were also a common theme across studies and included a dislike of drugs and long-term effects, dislike of non-natural treatments, perceived inefficacy of long-term medication and medication cost.	Medication-related concerns (perceived risk; dislike of medication; long term effects; non-natural treatments; perceived inefficacy of long-term medication)	Anticipated regret, information about health consequences, salience of consequences, credible source, framing/reframing
Anticipated and unanticipated and unpleasant side effects were frequently described.	Medication-related concerns (side effects; coping with side effects)	Social support (unspecified), social support (practical), social support (emotional), problem solving, pros and cons, information about health consequences
Patients worried about interactions between medications, and wanted to avoid feeling 'like you're rattling because I'm taking the whole blooming chemist'	Medication-related concerns (toxicity and polypharmacy)	Information about health consequences, anticipated regret, pros and cons, comparative imagining of future outcomes, credible source

Uncertainty about diabetes medication was linked to an overall dislike of Western medicines by people of Pakistani and Indian origin in one study.	Medication-related concerns (dislike of Western medication)	Information about health consequences, credible source anticipated regret, framing/reframing
The negative aspects of the medication-taking experience in Type 2 diabetes contributed to the active non-adherence described below, with patients more likely to take medications if perceived advantages outweighed disadvantages.	Perceived benefits	Pros and cons, comparative imagining of future outcomes, anticipated regret, framing/reframing
Theme: The passive patient as active experimenter		
The passive patient		
Discussion of medication concerns with providers was the specific focus of only one study but interactions with providers was a feature of many others. Al-Qazaz et al. identified a conflict between patient satisfaction with healthcare interactions and the limited provision of information.	Patient-physician communication and relationsihp<br><br>Health-related information (provision; improving knowledge)	Social support (practical; from GP?), information about health consequences, credible source (NHS)<br><br>Information about health consequences, credible source (NHS), social support (practical)
Lawton et al. suggested participants needed non-judgemental guidance on missed doses but felt unable to discuss adherence choices with providers.	Social support<br><br>Health-related information (provision; improving knowledge)	Social support (emotional), social support (practical), social support (unspecified)<br><br>Information about health consequences
Active experimentation		
Across studies, authors characterised full adherence to medications rare, with the timing of doses particularly likely to be modified.	Self-adjustment/experimentation of medication dose	Self-monitoring of behaviour, self-monitoring of outcome(s) of behaviour, behavioural experiments
The specific phrase 'deliberate and routine adjustments' was used in two studies to describe this particular form of non-adherence. Lawton et al. provided an illuminating participant quotation evidencing deliberate adjustment 'sometimes I will take two when I don't spread too much jam on my toast or even sometimes I don't spread any. If I feel like a bit of pleasure then I will put some on and take the extra tablets'.	Self-adjustment/experimentation of medication dose<br><br>Perceived behavioural control	Self-monitoring of behaviour, self-monitoring of outcome(s) of behaviour, behavioural experiments<br><br>Information about antecedents, re-attribution
Alongside meal size and content other reasons for dose adjustments included to	Self-adjustment of medication dose	Self-monitoring of behaviour, self-

reduce unpleasant side effects, dislike of multiple medications, self-monitoring of blood glucose, with tablets used only when blood glucose was perceived to be high, …	 <br>Coping with side effects<br><br>Medication-related concerns (toxicity)<br><br>Self-monitoring (of outcomes)	monitoring of outcome(s) of behaviour, anticipated regret<br><br>Information about health consequences, social support (practical), social support (emotional) problem solving, anticipated regret<br><br>Information about health consequences, credible source, social support (emotional)<br><br>Self-monitoring of outcome(s) of behaviour
…a belief that medication provide symptomatic relief and are not needed when feeling well, preference for traditional medicines,…	Necessity-beliefs (medication not needed when asymptomatic)<br><br>Perceived seriousness	Self-monitoring of outcome(s) of behaviour, comparative imagining of future outcomes, anticipated regret, framing/reframing, pros and cons, feedback on outcomes of behaviour<br><br>Anticipated regret, framing/reframing, pros and cons
…lack of trust in providers…	Attitude (distrust of health care providers)	Anticipated regret, framing/reframing, pros and cons
…and to fit with personal understanding of the body's needs.	Necessity-beliefs (perceived health/self-monitoring)	Self-monitoring of behaviour, self-monitoring of outcome(s) of behaviour, information about health/emotional/social/ environmental consequences
Reducing medication in line with fasting during Ramadan was mentioned in three studies and could lead to a belief that medication intake could be reduced on other occasions.	Self-adjustment/experimentation with medication dose	Problem solving, generalisation of a target behaviour, self-monitoring of behaviour, self-monitoring of outcome(s) of behaviour
The active experimenter engaged in personal experiments to search for proof that	Self-monitoring of outcome(s) of	Self-monitoring of behaviour, self-

medications were working; symptom reduction and blood glucose readings were used to evidence the impact of medication. Patients also continued to take medications if negative effects were experienced when medications were discontinued.	behaviour<br><br>Self-adjustment/experimentation<br><br>Response efficacy	monitoring of outcome(s) of behaviour, behavioural experiments, feedback on outcomes of behaviour<br><br>Self-monitoring of behaviour, self-monitoring of outcome(s) of behaviour, behavioural experiments<br><br>Information about health consequences, feedback on outcomes of behaviour, credible source
Lawton et al. provided some of the strongest evidence of personal experiments under their theme evidencing the impact, and described that without obtaining their own proof, patients may be sceptical about the benefits of medication.	Perceived benefits<br><br>Medication-related concerns (scepticism about benefits of medication/drug efficacy)	Information about health consequences, framing/reframing, credible source, pros and cons<br><br>Behavioural experiments, information about health consequences, salience of consequences, anticipated regret, credible source
The active nature of adherence was also evident in the personal responsibility for medication described across studies; participants described personal guilt and concern about health consequences if non-adherent	Negative emotions	Framing/reframing, reduce negative emotions, information about emotional consequences, re-attribution, verbal persuasion about capability
Although support from family and friends was described in two studies, adherence was chiefly regarded as the patient's own responsibility. Medication adherence was described as 'down to me', patients were described as self-dependent and medication-taking during Ramadan was described as a private issue in one study.	Self-efficacy<br><br>Perceived behavioural control<br><br>Social support	Verbal persuasion about capability, social reward<br><br>Mental rehearsal of successful performance, focus on past success, social support (unspecified), verbal persuasion about capability<br><br>Social support (unspecified), social support (practical), social support (emotional)
A potential contradiction to the actively experimenting patient concept came from the	Health-related knowledge	Information about health consequences

discussion of forgetting or lack of knowledge as a major cause of non-adherence, with broken routines a frequently described cause of forgetting.	Necessity beliefs (forgetting / lack of motivation)<br><br>Habits (routine)	Anticipated regret, framing/reframing<br><br>Habit formation, action planning
Strategies used by patients to improve adherence, including developing routines and the use of prompts and reminders, suggested that the impact of forgetting on adherence could be minimised when patients were truly motivated.	Prompts and reminders<br><br>Habits (routine)<br><br>Motivation (necessity beliefs)	Prompts/cues, adding objects to the environment, restructuring the physical environment<br><br>Habit formation, behavioural practice/rehearsal<br><br>Framing/reframing, self-talk, anticipated regret, salience of consequences
This issue was openly addressed by Al-Qazaz et al. who argued that, although forgetting has traditionally been classified as non-intentional non-adherence, it can also be understood as an indication of limited belief in medication necessity.	Necessity beliefs (forgetting to take medication)	Information about health consequences, salience of consequences, anticipated regret, pros and cons, comparative imagining of future outcomes
*Theme: Taking oral medication for Type 2 diabetes: a unique context**		
Most participants described negative reactions to oral medication initiation, felt uneasy about medication use and wanted to stay diet controlled for as long as possible. Initiation of oral medication was described as 'a slippery slope' of increasing medication, which would eventually lead to insulin. Lawson et al. also linked initiation to identity change as 'if you start taking them, you become a patient'. Initiation could be seen as personal failure, indicating that the patient had been unable to manage diabetes themselves.	Identity<br><br>Self-efficacy<br><br>Perceived susceptibility	Valued self-identity associated with changed behaviour, incompatible beliefs<br><br>Verbal persuasion about capability, mental rehearsal of successful performance, focus on past success, self-talk, social support (emotional), social reward<br><br>Pros and cons, re-attribution,

		comparative imagining of future outcomes
Two particular features of type 2 diabetes appeared to have an impact on taking medication: the asymptomatic nature of diabetes and the perceived relationship between medication, diet, and glucose control. The asymptomatic nature of diabetes, resulting in the condition not being ‘at the forefront of your mind’, was identified as a reason for non-adherence in three studies. Patients were described as having a high awareness of the relationship between food intake and medication and deliberate and routine dose adjustment often went hand in hand with dietary choices. This link with diet resulted in patterns of non-adherence particular to the diabetes context, with patients taking extra tablets after a heavy meal or skipping a tablet if a meal was missed.	Self-adjustment of medication dose<br><br>Perceived behavioural control<br><br>Necessity-beliefs (forgetting to take medication; asymptomatic nature of diabetes; impact of dietary choices and self-adjustment)	Re-attribution, self-monitoring of behaviour<br><br>Framing/reframing, re-attribution, anticipated regret<br><br>Habit formation, behavioural practice/rehearsal, framing/reframing, pros and cons, information about health consequences

*”The final third-order construct describes the features of medication-taking which are specific to type 2 diabetes. A contrast between experiences of taking type 2 diabetes medications and other medications was not a specific theme identified in any of the included studies. The development of this third-order construct was informed by considering identified translations in relation to the review team’s knowledge of, and engagement with, the medication-taking literature more broadly.” McSharry et al.

Table 2. Brundisini et al. (2015)

Quote	Construct	Possible BCTs
Theme: Emotions increasing and decreasing adherence		
Positive emotions, such as experiencing positive health beliefs of insulin treatment, can reinforce self-reported feelings of empowerment, and the ability to follow-through with self-care.	Self-efficacy	Monitoring of emotional consequences, verbal persuasion about capability, self-talk, mental rehearsal of successful performance, focus on past success
Emotional and social support promotes a sense of self-efficacy and commitment to lifestyle changes, encouraging patients to do better to stay ‘on track’.	Social support<br><br>Self-efficacy	Social support (emotional), social support (practical),<br><br>Verbal persuasion about capability, social reward, self-talk, information about others’ approval

Negative emotions such as fear, self-blame, guilt, shock, helplessness and frustration can also either raise or lower adherence.	Self-efficacy<br><br>Social support<br><br>Negative emotions	Verbal persuasion about capability, self-talk, focus on past success, mental rehearsal of successful performance<br><br>Social support (emotional), social support (practical)<br><br>Reduce negative emotions, social support (emotional)
Patients frightened by symptoms returning, early death, and potential complications of diabetes sometimes become more serious about medication adherence.	Perceived seriousness (of diabetes)<br><br>Perceived susceptibility (to diabetes worsening)<br><br>Perceived benefits (of medication)	Anticipated regret, information about health/emotional/social/ environmental consequences<br><br>Comparative imagining of future outcomes, info about health consequences, anticipated regret<br><br>Information about health/emotional/social/ environmental consequences, pros and cons
Observing the suffering of others with diabetes complications can motivate patients to adhere strictly to their own treatments.	Social comparison	Anticipated regret, comparative imagining of future outcomes, social comparison
Some patients prefer providers to emphasise the potential benefits of adhering, rather than the risk of non-compliance.	Perceived benefits (of medication)	Comparative imagining of future outcomes, pros and cons, information about health consequences, credible source
Those with increasing complications and intensifying treatment sometimes feel they have already failed at managing the disease, creating a 'vicious circle of low motivation'.	Medication concerns (regimen complexity)<br><br>Self-efficacy	Framing/reframing, habit formation, prompts and cues, problem solving, adding objects to the environment<br><br>Verbal persuasion about capability, mental rehearsal of successful performance, focus on past success, self-talk, reduce negative emotions,

		feedback on outcomes of behaviour
Distress – whether from diabetes or other sources – can also demotivate medication adherence.	Negative emotions (distress)	Monitoring of emotional consequences, reduce negative emotions, information about emotional consequences, social support (emotional)
Comorbid conditions such as heart disease, hypertension, depression, kidney failures, and decreasing sight can also lead to stress and complicate self-management practices.	Regimen complexity<br><br>Negative emotions<br><br>Self-efficacy	Habit formation, prompts and cues, conserving mental resources, problem solving<br><br>Reduce negative emotions, social support (emotional)<br><br>Verbal persuasion about capability, mental rehearsal of successful performance, focus on past success, self-talk
Comorbidities can have the opposite effect of increasing motivation as successful self-management promotes self-confidence.	Self-efficacy	Mental rehearsal of successful performance, focus on past success, self-talk, verbal persuasion about capability, feedback on outcomes of behaviour
Theme: Intentional non-adherence		
Patients' beliefs and attitudes toward the health care system can promote informed and not informed intentional non-compliant behaviours. Intentional non-adherence sometimes results from denial about the seriousness of diabetes.	Perceived seriousness (denial)<br><br>Necessity-beliefs (denial)<br><br>Attitudes	Anticipated regret, framing/reframing, information about health/emotional/ social/environmental consequences, feedback on outcomes of behaviour<br><br>As above<br><br>Framing/reframing

Denial of the severity of diabetes may relate to the belief that ‘everybody’s got it’, or to the underlying scepticism and lack of trust about the effectiveness of the treatment coupled with the fear that the prescribed medication is unnecessary, unhealthy or dangerous.	Perceived seriousness (denial)<br><br>Necessity beliefs (denial)<br><br>Medication-concerns (distrust in drug efficacy)<br><br>Response efficacy	Framing/reframing, information about health/emotional/social/ environmental consequences, anticipated regret, feedback on outcomes of behaviour<br><br>As above<br><br>Pros and cons, information about health consequences, feedback on outcomes of behaviour<br><br>As above
Most commonly patients decide not to adhere to medication regimens as an effort to avoid side effects. This type of intentional non-compliance often takes a trial and error approach, with the patient self-adjusting medication (i.e. doses and timings).	Coping with side effects (self-adjustment/experimentation)	Anticipated regret, information about health consequences, problem solving
Theme: Patient-provider relationship and communication		
Many patients remark on the disconnect between treatment recommendations and their everyday life, as well as perceptions of lack of support, communication barriers, challenges of working with culturally insensitive providers, and barriers to accessing health care providers, such as time constraints during visits.	Tailoring (lack of)<br><br>Social support (lack of)<br><br>Perceived barriers (communication; time constraints during appts; cultural insensitivity)	Graded tasks, action planning, information about health consequences, problem solving<br><br>Social support (emotional, practical, unspecified)<br><br>Problem solving, social support (practical)
Patients describe a desire to be ‘perceived as persons, not illnesses’	Identity	Valued self-identity, identification of self as role model, identity associated with changed behaviour

Without the understanding of patient's life contextual factors, providers may set unrealistic targets, which patients deem impossible, thus frustrating.	Tailoring (lack of)<br><br>Self-efficacy<br><br>Negative emotions (frustration)	Graded tasks, action planning, information about health consequences<br><br>Focus on past successes, mental rehearsal of successful performance, verbal persuasion about capability<br><br>Reduce negative emotions, social support (emotional)
Patients attribute providers' unrealistic expectations to a lack of support or disinterest, which results in feelings of distrust. Many patients report hope for a collaborative relationship based on mutual trust and agreement between patient and provider, which would allow them to openly discuss their challenges and concerns with the providers.	Social support (lack of)<br><br>Perceived barriers (distrust of providers, patient-provider communication, provider support)	Social support (practical, emotional, unspecified)<br><br>Credible source (NHS), problem solving, information about health consequences
Both non-marginalised and socially and culturally marginalised patients, such as indigenous groups, immigrants, and visible minorities stress the importance of their relationship with the provider. Although each population group focuses on different aspects of such relationships, both groups place great value on the patient-physician relationship as a beneficial factor for medication adherence.	Patient-physician relationship/communication (importance of)	Credible source, social support (practical/emotional - from a credible source?)
Patients consider providers the major and most reliable source of information about their condition and their treatment.	Health-related information	Credible source (NHS/HP)
Communication barriers may inhibit collaborative relationships, preventing a shared understanding of treatment and therefore hindering medication adherence.	Perceived barriers (communication) (to improving knowledge)	Information about health consequences, credible source, social support (unspecified)
Authors describe the barriers to communication between patients and providers as reflecting differences in underlying health beliefs and different desires and understandings of the model of care. Patients attribute communication barriers to the way clinicians communicate with them, including providing information that is ambiguous, incomplete, or irrelevant, provider time constraints, and lack of shared decision-making strategies among multiple health care providers.	Perceived barriers (communication barriers, time constraints in healthcare, unclear health information)<br><br>Tailoring (lack of)	Information about health consequences, salience of consequences<br><br>Graded tasks, action planning, problem solving, information about health consequences

Information inconsistency and lack of clear information may result in misunderstandings, and lead patients to use other sources of information.	Health-related information/knowledge	Credible source, information about health consequences, salience of consequences
Theme: Information and knowledge		
Patients accounts of how they negotiate their medication regimens offers explanations for why they choose to manage their condition in a way that suits their personal circumstances and understanding of their health, body, and diabetes.	Self-adjustment/experimentation with medication	Self-monitoring of behaviour, self-monitoring of outcome(s) of behaviour, behavioural experiments, information about health consequences
For some patients, a lack of understanding and inadequate knowledge about the medication and prevention of a complication results in poorly controlled blood glucose levels and poor adherence.	Health-related knowledge	Instruction on how to perform the behaviour, re-attribution, information about health consequences, salience of consequences, action planning
In other instances, patients report that they understood medications' importance, but not how the medications work.	Necessity-beliefs (medication is important)<br><br>Health-related knowledge	Information about health/social/environmental/ emotional consequences, salience of consequences<br><br>Information about health/social/environmental/ emotional consequences, salience of consequences
Patients often report abstaining from medication when asymptomatic, or they consciously decide to take medication according to how they feel.	Self-adjustment/experimentation with medication<br><br>Self-monitoring (of symptoms)	Behavioural experiments, self-monitoring of behaviour, self-monitoring of outcome(s) of behaviour<br><br>Self-monitoring of behaviour, self-monitoring of outcome(s) of behaviour
Other studies describe patients as knowledgeable, but unable to translate this knowledge into appropriate action (e.g. 'what to do when things go wrong'). This kind of understanding may improve with experience, necessitating a set of problem solving strategies, including creative solutions to diabetes self-management.	Coping with side effects<br><br>Problem solving<br><br>Self-management (strategies and	Problem solving, re-attribution, information about health consequences<br><br>Problem solving, action planning<br><br>Habit formation, behavioural practice/rehearsal, graded tasks,

	solutions)	generalisation of a target behaviour
Patients value the information received by their providers on medication treatment, self-management strategies and on navigating the health care system. Patients also value the information provided by a variety of ancillary resources, such as clinic dieticians and nutritionists and peer support groups and educational programmes including self-management education classes and medication counselling services.	Health-related knowledge<br><br>Self-management (strategies and solutions)<br><br>Social support	Credible source(s), information about health/emotional/social/ environmental consequences<br><br>Habit formation, behavioural practice/rehearsal, graded tasks, generalisation of a target behaviour<br><br>Social support (emotional), social support (practical), social support (unspecified) information about others' approval, social comparison
Patients note that they appreciate the opportunity to share information and knowledge and learn from others who live with the same condition who successfully cope with their condition.	Health-related knowledge<br><br>Social comparison/influence<br><br>Social support	Credible source(s), information about health/emotional/social/ environmental consequences<br><br>Social comparison, information about others' approval, social support (emotional), social support (practical), social support (unspecified)<br><br>Social support (practical), social support (emotional), social support (unspecified)
Patients also identify the need or desire for more information and management strategies, especially in language and culture-specific ways.	Health-related knowledge<br><br>Self-management (strategies)	Instruction on how to perform the behaviour, information about health consequences, salience of consequences, social support (practical)<br><br>Habit formation, behavioural practice/rehearsal, graded tasks,

		generalisation of a target behaviour
Theme: Medication administration		
For many patients, the administration of medication poses a significant barrier to adherence. Patients describe fear as a common barrier to insulin administration, in particular fear of needles, fear of consequences of administering insulin incorrectly, and the pain of injection or blood testing.	Medication-related concerns (barriers to medicine administration; fear of administering insulin correctly, needles, testing blood glucose)<br><br>Perceived barriers (medicine administration)	Reduce negative emotions, information on how to perform the behaviour, demonstration of the behaviour, information about health consequences<br><br>As above
Patients relate insulin administration to other psychological barriers, such as a feeling of stigma around the possession and use of an injectable medication because of the possibility of being mistaken for an illicit drug.	Perceived barriers (psychological barriers of stigma; social influence)	Identification of self as role model, valued self-identity, information about antecedents, framing/reframing
Comorbid conditions represent a general barrier to medication administration. Patients who take multiple medications may experience forgetfulness, confusion about the purpose, name, and the potential for interactions with other medications. The burden of the medication regimen is typically linked to the rigidity of medication which impedes flexibility in everyday life, as this patient reports, ‘just the timing and remembering to take your pills on time. It’s a real effort to take them at the right time’.	Medication-related concerns (regimen complexity; forgetfulness/confusion; risk of toxicity)<br><br>Perceived barriers (burden of regimen complexity)<br><br>Health-related knowledge	Prompts/cues, problem solving, habit formation, conserving mental resources<br><br>Conserving mental resources, habit formation, prompts/cues<br><br>Information about health consequences, credible source
The development of habit-forming routines may encourage medication adherence. When the patient hasn’t established, or has disrupted, the routine, medication adherence declines. This includes minor (e.g. skipping meals), or social and contextual factors in the patient’s life, such as childcare, domestic duties, or work schedules can interfere with patient’s routines.	Habit formation	Habit formation, habit reversal, prompts and cues, behavioural practice/rehearsal, generalisation of a target behaviour, graded tasks
Patients acknowledge family’s instrumental support as a practical means to help integrate the treatment regimen in patients’ daily lives. However, some patients describe fear of being a burden on their family or unsupportive family members as a direct barrier to medication adherence.	Social support (practical)<br><br>Habit formation<br><br>Perceived barriers	Social support (practical)<br><br>Habit formation<br><br>Valued self-identity, restructuring the social environment, information about

		social and environmental consequences, information about antecedents, avoidance/reducing exposure to cues for the behaviour
Theme: Social and cultural health beliefs		
Health beliefs about medication and diabetes are often linked to social or cultural understanding about the body, diabetes and medication, which in turn can affect medication adherence in many different ways. Multiple factors shape these health beliefs, such as the information sources used by the patient, past experiences, attitudes of others, faith and religious beliefs, education, and cultural community. A patient's health belief system may affect the way he or she describes to approach medication adherence, and how to integrate (or not) the requirements of the medication regimen into everyday life.	Health-related information<br><br>Social support (influence)<br><br>Attitudes (and beliefs)	Credible source, information about health consequences<br><br>Social support (practical), social support (emotional), social support (unspecified), information about antecedents<br><br>Framing/reframing, information about health/emotional/social/ environmental consequences, anticipated regret, pros and cons
A patient's health beliefs and cultural background will also affect the relationship s/he desires with the prescribing physician. For instance, patients who are members of historically oppressed communities by the dominant culture can be suspicious of medical advice. Patients from cultures that perceive physicians as high status individuals with significant authority may feel uncomfortable asking questions. Several papers recommend including the patient in a culturally appropriate way as an active partner of care to improve medication adherence. However, providers should adapt to the patients' beliefs and preference, as some patients may refuse to work with clinicians in this way.	Perceived barriers (status/authority of doctor; patient-provider relationship)<br><br>Medication-related concerns (distrust of Western drs/health advice)	Framing/reframing, information about others' approval<br><br>Anticipated regret, information about health/emotional/social/ environmental consequences
Social and cultural beliefs also affect patient preferences for allopathic (Western biomedicine) compared to traditional medications. Some patients express their intention to take them alongside traditional medications. Many patients indicate a preference for traditional or herbal medications, and a suspicion or distrust of allopathic medication. These patients view allopathic medicine as unnatural, incongruent with their understanding of holistic health. In contrast, patients describe traditional remedies as effective, a link to their past and present cultural communities and easier to access.	Medication-related concerns (distrust of Western medication; response efficacy of traditional herbal medicines vs. Western medicines; medication is unnatural)	Information about health consequences, feedback on outcomes of behaviour

Table 3. Kumar et al. (2016)

Quote	Construct	Possible BCTs
Theme: beliefs about the need for and efficacy of medication		
Many patients of South Asian origin regarded medicines for the treatment of diabetes and CVD as necessary […] Patients also recognised it was necessary to take these medicines on a long term basis.	Necessity beliefs (believed medication necessary; long-term)	Information about health consequences
The danger of not taking medicines was recognised and the roles of medicines in reducing the risks associated with illness were described.	Perceived benefits (of medication)<br><br>Response efficacy	Anticipated regret, pros and cons<br><br>Information about health consequences, self-monitoring of outcome(s) of behaviour, credible source, feedback on outcomes of behaviour
Not only were medicines seen as necessary but they were also viewed as effective. However, the perceived effectiveness of medicines varied depending upon where they had been prescribed. Patients who had migrated to the UK described the medicines they received in the UK as being more effective than those they would have received in places like India and Pakistan. These pro-western medicine thoughts were seen also in those who also tried alternative medicines. Patients appeared to place a high value on evidence based medicines prescribed in the West. However, traditional and alternative medicines that originated from the South Asian region were also valued, and held by some in the same regard as medicines prescribed in the West.	Perceived benefits (of both Western and traditional medicines)<br><br>Response efficacy	Incompatible beliefs, framing/reframing, information about health consequences<br><br>Information about health consequences, self-monitoring of outcome(s) of behaviour, anticipated regret, feedback on outcomes of behaviour, credible source
While some spoke of the need for their medicines, many patients reported having missed doses of their medicines intentionally. In some cases, somatic cues such as “feeling fine” were given as reasons for not taking medicines […] Some adjusted their diabetic medicine according to what they ate. Some patients made a conscious decision to stop their treatment during social gatherings. South Asian patients often stopped their medicines to fully partake in activities such as weddings.	Self-adjustment of dose/experimentation with dose<br><br>Self-monitoring (of symptoms/dietary intake)	Anticipated regret, framing/reframing, self-monitoring of behaviour, self-monitoring of outcome(s) of behaviour<br><br>Self-monitoring of behaviour, self-monitoring of outcome(s) of behaviour, information about health consequences<br><br>Restructuring the social environment,

	Social support (influence)	generalisation of a target behaviour, avoidance/reducing exposure to cues for the behaviour
In some cases, patients were influenced by other family members' experiences of taking medicines.	Social support (influence)	Social support, social comparison, information about others' approval, credible source, demonstration of behaviour
Some patients felt that the complications of their illness were unavoidable and inevitable even if medicines were taken.	Perceived seriousness<br><br>Perceived benefits (lack of)<br><br>Self-efficacy	Framing/reframing, pros and cons, information about health consequences<br><br>Anticipated regret, information about health/emotional/social/ environmental consequences, pros and cons, comparative imagining of future outcomes, feedback on outcomes of behaviour<br><br>Verbal persuasion about capability, mental rehearsal of successful performance, focus on past success, self-talk, social support (emotional)
A few patients used self-management strategies and life style changes as a rationale for stopping their medicine.	Self-adjustment/experimentation	Anticipated regret, information about health consequences, pros and cons, comparative imagining of future outcomes, framing/reframing
Theme: toxicity of medicines and polypharmacy		
Some patients were concerned about increasing numbers of prescribed medicines being added to their treatment regimens, adding to their fears of toxicity.	Medication concerns (regimen complexity; toxicity and polypharmacy)	Conserving mental resources, information about health consequences, framing/reframing, self-monitoring of outcome(s) of behaviour, feedback on outcomes of behaviour, social support (unspecified)
Concerns were expressed about potential side effects of medicines, and beliefs about	Medication concerns (side effects;	As above

toxicity (with medicines viewed as poisons). A number of patients feared that taking too many medicines would lead to death.	toxicity; risk of death)	
South Asian patients suggested that taking too many medicines caused them to feel “dull” and “dry”.	Medication concerns (side effects)	Information about health consequences, framing/reframing, reducing negative emotions
In instances where toxicity had been experienced, anger was expressed. Patients felt frustrated when one tablet resulted in a complication to control which they had to take an additional medicine.	Negative emotions	Reducing negative emotions, social support (emotional)
Theme: stigma and social support		
Stigma and social support had a major influence on medicine taking. Patients were reluctant to disclose their use of insulin to their families and community. Some patients were told by family members not to tell anyone that they were taking insulin, which made it difficult to take the medication during social occasions.	Social support (influence and stigma)<br><br>Identity	Restructuring the social environment, social support, avoidance/reducing exposure to cues for the behaviour, information about antecedents<br><br>Valued self-identity, identification of self as role model, identity associated with changed behaviour
In some cases, coping strategies were developed to ensure that the insulin was not used during social situations, including taking insulin before attending the social function.	Social support (influence and stigma)	Restructuring the social environment, avoidance/reducing exposure to cues for the behaviour
The social stigma attached to diabetes and insulin therapy was associated with embarrassment on the part of the patient. This made some patients reluctant to initiate insulin therapy. Patients felt that illnesses such as diabetes were not socially acceptable within South Asian communities. For people from a South Asian background, diabetes and insulin were viewed in very negative terms, and it was expressed that they were not culturally accepted.	Social support (stigma)<br><br>Identity	Information about others’ approval, restructuring the social environment, information about antecedents, incompatible beliefs<br><br>Valued self-identity, identification of self as role model
In contrast, in some situations close family were felt to be positive agents in supporting self-management and often helped to facilitate medicine taking. Some patients had a family support mechanism which they felt helped them to take medicines.	Social support	Social support (practical), social support (emotional), social support (unspecified)
Theme: the necessity of traditional remedies versus Western medicines		
Patients experimented with traditional remedies in parallel with Western medicines.	Medication-related concerns (toxicity	Anticipated regret, self-monitoring of

Many patients made use of traditional remedies alongside their Western medicines and did not associate “herbal” medicines with the perceived toxicity associated with medicines prescribed by the doctor. Not only were traditional remedies viewed as necessary, they were also viewed as effective.	of Western medication)<br><br><br>Response efficacy	outcome(s) of behaviour, framing/reframing<br><br>Anticipated regret, information about health consequences, credible source, feedback on outcomes of behaviour
Patients also described a number of positive attributes of traditional remedies, including the fact that these remedies “make quite a difference”, have no side effects, provide balance, and are natural. In some cases patients described traditional remedies as being better at tackling illnesses and able to control the adverse consequences of illness.	Response efficacy	Anticipated regret, information about health consequences, credible source, feedback on outcomes of behaviour
Media within the South Asian community often portrayed these remedies in a positive light. Family and friends were important in decisions to use alternative medicines, and in some cases would supply these medicines.	Social influence	Information about health consequences, anticipated regret, restructuring the social environment, credible source, avoidance/reducing exposure to cues for the behaviour
Some patients placed more faith in Western medicines than traditional remedies. One patient spoke of how they had moved away from traditional remedies to accept medicines prescribed by the doctor, while others spoke of how traditional remedies played a large role in the management of diabetes in South Asian countries.	Medication-related concerns (traditional vs. Western medications	Credible source, anticipated regret, information about health consequences
Theme: communication		
Health professionals’ communication styles influenced the way patients viewed the disease process and medicines. Some patients felt that they were not always fully informed about disease management and how the medicines would help to control their symptoms. Some expressed a lack of engagement with the decisions that many doctors made and did not understand the treatment plan.	Patient-provider communication<br><br><br><br>Health-related knowledge	Information about health consequences, pros and cons, framing/reframing<br><br><br>Information about health consequences
Patients often compared and contrasted different health systems: for example, patients felt that the healthcare system in the UK was more trustworthy than countries such as India and Pakistan. This view had an impact on the way patients communicated with UK doctors about their medicines, and followed advice.	Credible source	Credible source (NHS)

Table 4. Ng et al., 2015 – barriers and facilitators to starting insulin in patients with type 2 diabetes

Quote	Construct	Possible BCTs
Theme: patient-related factors (insulin related factors)		
Eighteen studies reported fear of needle, injection and pain.	Medication-concerns (fear of insulin administration)	Behavioural practice/rehearsal, reduce negative emotions, monitoring of emotional consequences, social support (practical), social support (emotional)
Concern about side effects of insulin, such as hypoglycaemia and weight gain, was cited as a barrier in 12 studies.	Medication-concerns (side effects of medications)	Information about health consequences, pros and cons, comparative imagining of future outcomes, self-monitoring of outcome(s) of behaviour
Insulin administration was perceived by patients to be difficult and complicated. Some patients found it hard to adjust insulin dosages, some viewed home monitoring of blood glucose as an additional burden and more painful than insulin injections.	Medication-concerns (regimen and administration complexity; pain of administration; burden of blood glucose monitoring)	Demonstration of the behaviour, framing/reframing, information about health consequences, pros and cons, social support (practical), social support (emotional), social support (unspecified)
Insulin treatment was viewed as inconvenient to patients because they have to carry insulin pen with them and inject regularly.	Medication-concerns (burden and complexity of regimen)	Pros and cons, information about health consequences, restructuring the physical environment
In terms of facilitators, patients who are not afraid of injections and believe that insulin can help improve their blood sugar control, prolong their life, reduce complications and symptoms and is more effective than oral medications, were more likely to start insulin.	Necessity-beliefs (insulin improves health; insulin better than oral medication)	Information about health consequences, pros and cons, framing/reframing
Having undergone hands-on demonstration of insulin injection was also a facilitator to starting insulin.	Demonstration of the behaviour	Demonstration of the behaviour
Patient-related factors (personal factors)		
Thirteen studies reported negative perceptions on insulin therapy. To patients, insulin initiation signified serious stage of diabetes and considered as a last resort. Some were sceptical about the benefits of insulin while others perceived insulin as unclean, meant for older people and associated with sexual dysfunction in men.	Medication-concerns (scepticism of insulin efficacy; stigma)	Information about health consequences, framing/reframing, pros and cons, anticipated regret, credible source
Ten studies reported negative attitudes towards diabetes and its treatment which prevent them from starting insulin. Patient’s perceived starting insulin as a	Necessity-beliefs (no need to start insulin)	Information about health consequences, pros and cons,

punishment and personal failure. They also did not believe that there was a need to start insulin.	 <br>Self-efficacy<br><br>Perceived seriousness	anticipated regret, comparative imagining of future outcomes<br><br>Valued self-identity, identification of self as role model<br><br>Information about health consequences, pros and cons, anticipated regret, comparative imagining of future outcomes
Other negative attitudes include denial towards diabetes, inaccurate perceptions of treatment goal and fatalistic view about diabetes.	Perceived seriousness<br><br>Perceived susceptibility	Information about health consequences, pros and cons, comparative imagining of future outcomes<br><br>Anticipated regret, pros and cons, comparative imagining of future outcomes
Elderly patients were hesitant to initiative insulin if they had poor vision, unsteady hands or arthritis, and if they had little or no social support.	Social support	Social support (practical), social support (emotional), social support (unspecified)
Some patients preferred to use other treatment options including complementary and alternative medicines.	Necessity beliefs (alternative medicines vs. evidence based medicines)	Information about health consequences, pros and cons, anticipated regret, comparative imagining of future outcomes, framing/reframing
On the other hand, patients who were involved in decision making, had poor physical health and perceived that they have no choice were more likely to start insulin.	Necessity beliefs (insulin is necessary)	Information about health consequences, salience of consequences, pros and cons
Patient-related factors (social factors)		
Seven studies indicated that stigma and discrimination are barriers to starting insulin.	Social support (influence and stigma)	Valued self-identity, identification of self as role model, information about others' approval, social support (unspecified), social support

		(emotional), social support (practical)
Other social barriers include interference with social and work activities, family's and peers' negative views and experiences, loss of independence, lack of social support, and affecting relationship with significant others.	Social support (influence)	Social support (practical, emotional, unspecified), restructuring the social environment
Social factors that facilitated insulin initiation include positive experiences of others and support from family, peers, and healthcare providers.	Social support<br><br>Social comparison	Social support (practical, emotional, unspecified), credible source, information about others' approval<br><br>Social comparison
