# Supplementary material for: Identifying Brief Message Content for Interventions Delivered via Mobile Devices to Improve Medication Adherence in People With Type 2 Diabetes Mellitus: A Rapid Systematic Review
Source: J Med Internet Res. 2019 Jan 9;21(1):e10421. doi: 10.2196/10421 (PMC6329430; doi:10.2196/10421)
Supplement: Multimedia Appendix 3 [file jmir_v21i1e10421_app3.pdf]

Multimedia Appendix 3

Table 1. Behaviour Change Technique definitions from the Behaviour Change Technique Taxonomy Version 1 (Michie et al., 2013).

**BCT category**[a]<br>BCT	**Definition**
**1. Goals and planning**	
1.2. Problem solving	Analyse, or prompt the person to analyse, factors influencing the behaviour and generate or select strategies that include overcoming barriers and/or increasing facilitators.
1.4. Action planning	Prompt detailed planning of performance of the behaviour (must include at least one of context, frequency, duration and intensity). Context may be environmental (physical or social) or internal (physical, emotional or cognitive).
**2. Feedback and monitoring**	
2.3. Self-monitoring of behaviour	Establish a method for the person to monitor and record their behaviour (s) as part of a behaviour change strategy.
2.4. Self-monitoring of outcome(s) of behaviour	Establish a method for the person to monitor and record the outcome(s) of their behaviour as part of a behaviour change strategy
2.7. Feedback on outcome(s) of behaviour	Monitor and provide feedback on the outcome of performance of the behaviour.
**3. Social support**	
3.1. Social support (unspecified)	Advise on, arrange or provide social support *(e.g. from friends, relatives, colleagues,' buddies' or staff)* or non-contingent praise or reward for performance of the behaviour. It includes encouragement and counselling, but only when it is directed at the behaviour.
3.2. Social support (practical)	Advise on, arrange, or provide practical help *(e.g. from friends, relatives, colleagues, 'buddies' or staff)* for performance of the behaviour.
3.3. Social support (emotional)	Advise on, arrange, or provide emotional social support *(e.g. from friends, relatives, colleagues, 'buddies' or staff)* for performance of the behaviour.
**4. Shaping knowledge**	
4.1. Instruction on ow to perform the behaviour	Advise or agree on how to perform the behaviour.
4.2. Information about antecedents	Provide information about antecedents (*e.g. social and environmental situations and events, emotions, cognitions)* that reliably predict performance of the behaviour.
4.3. Re-attribution	Elicit perceived causes of behaviour and suggest alternative explanations *(e.g. external or internal and stable or unstable).*
4.4. Behavioural experiments	Advise on how to identify and test hypotheses about the behaviour, its causes and consequences, by collecting and interpreting data.
**5. Natural consequences**	
5.1. Information about health consequences	Provide information (e.g. written, verbal, visual) about health consequences of performing the

	behaviour.
5.2. Salience of consequences	Use methods specifically designed to emphasise the consequences of performing the behaviour with the aim of making them more memorable (goes beyond informing about consequences).
5.3. Information about social and environmental consequences	Provide information (e.g. written, verbal, visual) about social and environmental consequences of performing the behaviour.
5.4. Monitoring of emotional consequences	Prompt assessment of feelings after attempts at performing the behaviour.
5.5. Anticipated regret	Induce or raise awareness of expectations of future regret about performance of the unwanted behaviour.
5.6. Information about emotional consequences	Provide information (e.g. written, verbal, visual) about emotional consequences of performing the behaviour.
**6. Comparison of behaviour**	
6.1. Demonstration of behaviour	Provide an observable sample of the performance of the behaviour, directly in person or indirectly e.g. via film, pictures, for the person to aspire to or imitate.
6.2. Social comparison	Draw attention to others' performance to allow comparison with the person's own performance.
6.3. Information about others' approval	Provide information about what other people think about the behaviour. The information clarifies whether others will like, approve or disapprove of what the person is doing or will do.
**7. Associations**	
7.1. Prompts/cues	Introduce or define environmental or social stimulus with the purpose of prompting or cueing the behaviour. The prompt or cue would normally occur at the time or place of performance.
**8. Repetition and substitution**	
8.1. Behavioural practice/rehearsal	Prompt practice or rehearsal of the performance of the behaviour one or more times in a context or at a time when the performance may not be necessary, in order to increase habit and skill.
8.3. Habit formation	Prompt rehearsal and repetition of the behaviour in the same context repeatedly so that the context elicits the behaviour.
8.4. Habit reversal	Prompt rehearsal and repetition of an alternative behaviour to replace an unwanted habitual behaviour.
8.6. Generalisation of target behaviour	Advise to perform the wanted behaviour, which is already performed in a particular situation, in another situation.
8.7. Graded tasks	Set easy-to-perform tasks, making them increasingly difficult, but achievable, until behaviour is performed.
**9. Comparison of outcomes**	
9.1. Credible source	Present verbal or visual communication from a credible source in favour of or against the behaviour.
9.2. Pros and cons	Advise the person to identify and compare

	reasons for wanting (pros) and not wanting to (cons) change the behaviour.
9.3. Comparative imagining of future outcomes	Prompt or advise the imagining and comparing of future outcomes of changed versus unchanged behaviour
**10. Reward and threat**	
10.4. Social reward	Inform that a verbal or non-verbal reward will be delivered if and only if there has been effort and/or progress in performing the behaviour.
**11. Regulation**	
11.2. Reduce negative emotions	Advise on ways of reducing negative emotions to facilitate performance of the behaviour.
11.3. Conserving mental resources	Advise on ways of minimising demands on mental resources to facilitate behaviour change.
**12. Antecedents**	
12.1. Restructuring the physical environment	Change, or advise to change the physical environment in order to facilitate performance of the wanted behaviour or create barriers to the unwanted behaviour (other than prompts/cues, rewards and punishments).
12.2. Restructuring the social environment	Change, or advise to change the social environment in order to facilitate performance of the wanted behaviour or create barriers to the unwanted behaviour (other than prompts/cues, rewards and punishments).
12.3. Avoidance/ reducing exposure to cues for the behaviour	Advise on how to avoid exposure to specific social and contextual/physical cues for the behaviour, including changing daily or weekly routines.
12.5. Adding objects to the environment	Add objects to the environment in order to facilitate performance of the behaviour.
**13. Identity**	
13.1. Identification of self as role model	Inform that one's own behaviour may be an example to others.
13.2. Framing/reframing	Suggest the deliberate adoption of a perspective or new perspective on behaviour (e.g. its purpose) in order to change cognitions or emotions about performing the behaviour.
13.3. Incompatible beliefs	Draw attention to discrepancies between current or past behaviour and self-image, in order to create discomfort.
13.4. Valued self-identity	Advise the person to write or complete rating scales about a cherished value or personal strength as a means of affirming the person’s identity as part of a behaviour change strategy.
13.5. Identity associated with changed behaviour	Advise the person to construct a new self-identity as someone who ‘used to engage with the unwanted behaviour’.
**15. Self-belief**	
15.1. Verbal persuasion about capability	Tell the person that they can successfully perform the wanted behaviour, arguing against self-doubts and asserting that they can and will succeed.
15.2. Mental rehearsal of successful performance	Advise to practise imagining performing the behaviour successfully in relevant contexts.

15.3. Focus on past success	Advise to think about or list previous successes in performing the behaviour (or parts of it).
15.4. Self-talk	Prompt positive self-talk (aloud or silently) before and during the behaviour.

[a]
